# Evaluating the Anticholinergic Burden (ACB) of Patients Referred to Rotherham Memory Clinic

**DOI:** 10.1192/bjo.2025.10651

**Published:** 2025-06-20

**Authors:** Nkiruka Onyekwelu

**Affiliations:** Sheffield Health and Social Care Trust, Sheffield, United Kingdom

## Abstract

**Aims:** To evaluate if documentation of Anticholinergic burden (ACB) score is in accordance with the NICE guidelines.

To calculate the Anticholinergic burden (ACB) score for patients referred to memory clinic if not documented.

**Methods:** I conducted retrospective analysis using a systematic sampling method and a proforma on patient’s electronic medical record to ascertain if the ACBs of patients were documented when being reviewed.

Information was obtained from both SystmOne tabbed journals, SystmOne medications list, and referral letters (to determine ACB score documentation and calculation).

Two scoring systems were used to calculate ACB: ACB calculator (https://www.acbcalc.com/) and POMH data collection tool.

**Results:** Of the 92 patients referred to memory service, 30 patients were analysed and only 3 had documented ACB burden score (10%). Using the ACB scale. 13 individuals had ACB score of ≥2 which was 43% of patients analysed. Using POMH, 9 individuals had ACB score of ≥2 which was 30% of patients analysed.

Most common medication involved in individuals with ACB ≥3 was amitriptyline (67% using the POMH calculator and 37% using the ACB calculator) and all were commenced in primary care. If documented, this score would be classified as high risk and necessitate a medical review in line with the guidelines.

**Conclusion:** To regularly document Anticholinergic burden score for elderly patients referred to service.

Patients with high burden score may require a medication review with documented evidence of either: Discussion about reducing the dose; or stopping or switching the anticholinergic medicine.

Currently, no scoring system is recommended, to use an agreed system.

Reducing drugs with high ACB can also lead to less polypharmacy.

In addition, concomitant use with anticholinesterase inhibitors may reduce the effectiveness.

Reaudit second cycle in 6 months.

